# Climate Change and the Sea: A Major Disruption in Steady State and the Master Variables

**DOI:** 10.1021/acsenvironau.2c00061

**Published:** 2023-04-19

**Authors:** Reid A. Simmer, Emily J. Jansen, Kyle J. Patterson, Jerald L. Schnoor

**Affiliations:** † Department of Civil and Environmental Engineering, IIHR Hydroscience & Engineering, The University of Iowa, Iowa City, Iowa 52242, United States

**Keywords:** Climate Change, Ocean Chemistry, Climate Modeling, Master Variables, Carbon Dioxide, Ecosystems, pH, Coral Reef Decline

## Abstract

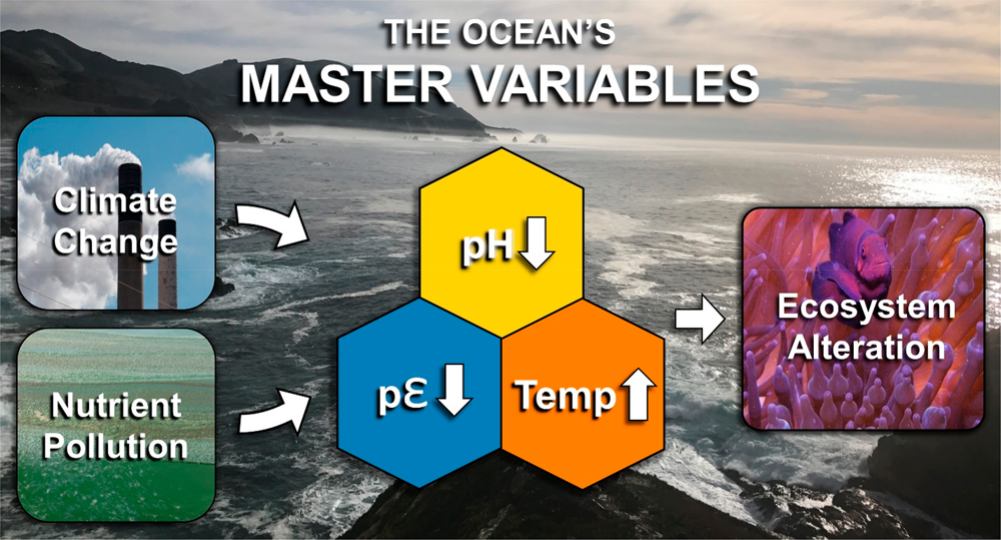

Since the beginning of the industrial revolution, humans have burned enormous quantities of coal, oil, and natural gas, rivaling nature’s elemental cycles of C, N, and S. The result has been a disruption in a steady state of CO_2_ and other greenhouse gases in the atmosphere, a warming of the planet, and changes in master variables (temperature, pH, and pε) of the sea affecting critical physical, chemical, and biological reactions. Humans have also produced copious quantities of N and P fertilizers producing widespread coastal hypoxia and low dissolved oxygen conditions, which now threaten even the open ocean. Consequently, our massive alteration of state variables diminishes coral reefs, fisheries, and marine ecosystems, which are the foundation of life on Earth. We point to a myriad of actions and alternatives which will help to stem the tide of climate change and its effects on the sea while, at the same time, creating a more sustainable future for humans and ecosystems alike.

## Introduction

A relatively steady climate has existed through the Holocene Period during the last 11,700 years, allowing humans to develop agriculture and cities. Prior to that, during the latter part of the Pleistocene Epoch (800,000 to 11,700 years ago), Earth’s climate underwent repeated glaciations every 100,000 years with widely varying temperatures (±4 °C), sea level (±6–9 m), and carbon dioxide (CO_2_) (from 290 to 190 ppm_v_) between interglacial and maximum glacial cycles.^1−5^

But now, the climate has changed rapidly during the current Anthropocene due to human activities, including burning fossil fuels, petrochemical production and release of methane gas, nitrogen fertilization of crops, and other forms of pollution. Here, we discuss changes in the steady state of the atmosphere and how that has affected master variables of chemistry and physics in the sea (pH, temperature, and oxidation–reduction potential or pε). Enormous quantities of coal, oil, and gas containing reduced compounds of C, N, and S were mined, combusted, and oxidized, thus, disrupting Earth’s elemental cycles, increasing greenhouse gases, warming the atmosphere, and transferring heat and CO_2_ into the sea.^6,7^

Driving forces of change during the Anthropocene include growth in human population, increasing per capita consumption, and certain polluting technologies. In 1950 the population was 2.5 billion, doubling to 5 billion in 1987. Today, the Earth’s population is 8 billion, an increase of 3 billion in the past 35 years. With more people on Earth, we have endeavored to provide food, water, housing, energy, transportation, infrastructure, and health care for all, resulting in burgeoning consumption. In fact, consumption and greenhouse gas emissions have increased far more rapidly than population alone, perhaps indicating an overall improvement in the human condition but at the expense of environmental preservation. According to the International Monetary Fund, the Gross World Product (GWP) of goods and services was approximately 4 trillion U.S. dollars in 1950 and rose to 94 trillion at the end of 2021–an increase greater than 23-fold in GWP, while the population only tripled.^8^ Human-caused CO_2_ emissions increased from about 5.5 billion metric tons (GtCO_2_) in 1950 to over 40 billion in 2020, a factor of roughly seven times.^9^ Unfortunately, some nations and some people consume far more than the planet can support.

Although greenhouse gases (GHGs) are trace constituents of the atmosphere, they are radiatively important trace gases (RITGs) that change the energy balance of planet Earth. Burning fossil fuels for energy, industry, heating, and transportation have released enormous quantities of greenhouse gases into the atmosphere, which warmed the air, Earth, and oceans resulting in a substantial change to master variables of the sea. In turn, changes in the master variables have caused dramatic effects on ice melting, sea level rise, coral reef decline, aragonite saturation, oxygen content, fisheries, and loss of ocean biodiversity.

In this review, we seek to explain climate change using simple chemical equilibrium and mass balance equations, together with figures illustrating the crux of the problem for the sea. Emphasizing the change in the steady state of master variables, we suggest solutions to mitigate climate change and preserve the ecology of the sea. This contribution is intended as a primer and a plea to environmental scientists for leadership on climate change. It is not a treatise but focuses on underappreciated changes in our vast and precious oceans.

## Massive Disruption in Elemental Cycles in Land/Atmosphere/Ocean

Since the beginning of the industrial revolution in the late 1700s, we have burned massive quantities of coal, oil, and natural gas to fuel everything from steam power to automobiles to assembly lines and computers. Energy acquired from burning fossil fuels allowed our economy to develop and provided a better way of life for billions of people on Earth. However, it has resulted in a massive disruption of Earth’s elemental carbon, nitrogen, and sulfur cycles. It is not that we are running out of fossil fuels. On the contrary, we have more than enough fossil fuels to supply human energy needs for centuries. However, we are running out of a place to store the exhaust from the combustion of coal, oil, and natural gas. Our exhaust is to the atmosphere, and it has been thrown out of a steady state and is accumulating greenhouse gases rapidly (exponentially). Because carbon dioxide and other greenhouse gases are mere “trace gases” in the atmosphere, it would seem that there should not be a problem. They alter the energy balance of the Earth, much like wrapping a blanket around the lower atmosphere (troposphere) and holding in the heat. Indeed, it results in “global warming,” but we call it *climate change* because there are so many manifestations other than rising temperatures causing problems on land and sea.

**Figure 1:**
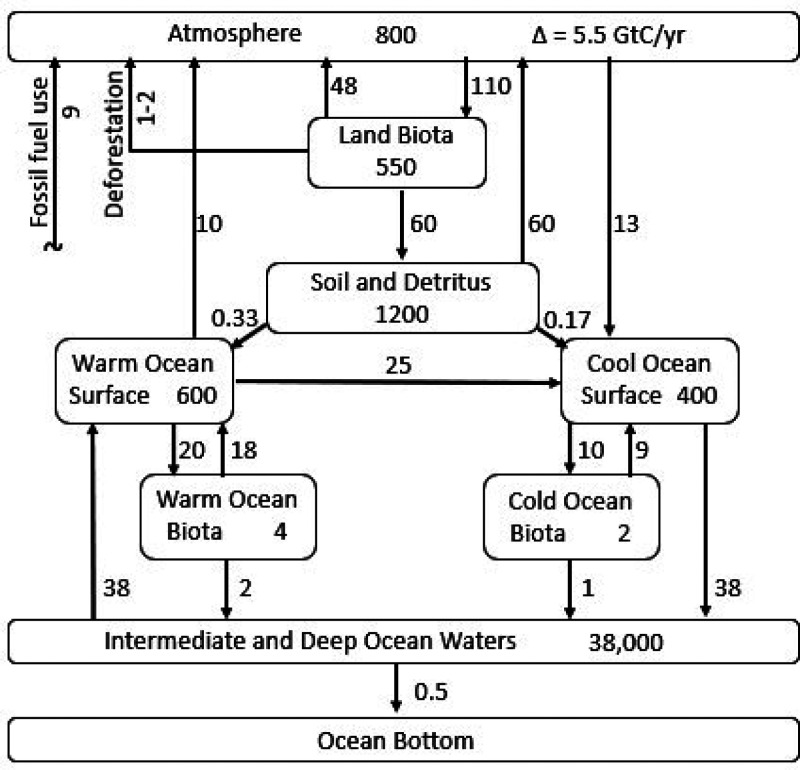
Global carbon box model schematic diagram. Units are gigatons of carbon (GtC) in reservoirs (numbers inside boxes) and gigatons as carbon per year (GtC/year) in fluxes (numbers by arrows). Adapted and updated with permission from ref (19). Copyright 1994 John Wiley & Sons, Inc.

Approximately 40 GtCO_2_ each year (10.9 GtC/year) are emitted due to fossil fuel combustion and land use change, such as deforestation (Figure 1). Carbon dioxide is by far the most dominant greenhouse gas, accounting for approximately 65% of the radiative effect. Methane, CH_4_, is the second most important greenhouse gas, accounting for 17% of the warming effect, followed by chlorofluorocarbon refrigerants (∼8%) and nitrous oxide, N_2_O, at 7% (Table 1).

**Table 1:** Major Greenhouse Gases (GHGs) Emitted to the Atmosphere Each Year by Humans, Their Concentration in the Atmosphere, Relative Contribution to Warming, Approximate Half-Life in the Atmosphere, and Their Radiative Importance Relative to Carbon Dioxide During a 100 Year Period (Adapted and Updated from Schnoor^10^)

greenhouse gas	preindustrial concentration (ppm_v_)^11^	2022 concentration (ppm_v_)^12^	atmospheric lifetime (years)^13^	global warming potential (GWP) value^14^
carbon dioxide (CO_2_)	280	416.77	variable	1
methane (CH_4_)	0.715	1.935	11.8	28
nitrous oxide (N_2_O)	0.27	0.336	109	265
CFC-11	0	0.000221	55	4600
CFC-12	0	0.000492	140	10200
HCFC-22	0	0.000248	13	1760
HCFC-134a	0	0.000120	<12	1300

Of the major fossil fuels, coal combustion emits the most CO_2_ per unit energy produced. It also emits nitrogen oxides (NO_*x*_) and sulfur oxides (SO_*x*_), major air pollutants. Eq 1 below shows an approximate stoichiometric chemical equation for the combustion of bituminous coal. Coal includes some ash and water, which further decreases its heating value compared to oil and natural gas. Diesel and low-S heating oil are represented by eq 2, and natural gas is represented by eq 3. (Natural gas is not pure methane as shown but contains varying amounts of ethane, propane, and other low molecular weight alkanes that cause the heating value to vary.) One can see from eqs 1–3 why coal is considered the “dirtiest” of fossil fuels. For an equivalent amount of heat (power) generated, coal emits roughly twice as much CO_2_ as natural gas. Conversely, as the equations show, for an equivalent amount of CO_2_ produced, natural gas provides twice the heat (energy) production as coal.^15,16^
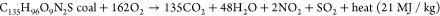
1
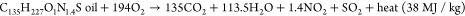
2
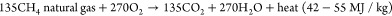
3

Coal was formed in the Earth’s crust from decaying vegetation over geologic time as early as the Carboniferous Age (300–360 million years ago). We mine fossil fuels which took millions of years to form in the Earth’s crust, burn them, and release carbon dioxide to the atmosphere in a couple of hundred years–just the blink of an eye in geologic time. So, it is no wonder that we have changed the chemical composition of the relatively small compartment on Earth that we call the atmosphere and disrupted the steady state.

At the Paris Climate Conference in 2015, 197 nations unanimously declared their intention to limit global warming to “well below 2 °C” and, furthermore, to engage in an effort to limit the temperature increase to not more 1.5 °C. The Global Carbon Project^17^ estimates that the remaining carbon budget to avoid warming of 1.5 °C is 420 GtCO_2_ as of 2020. Based on recent average annual emissions of 38.2 GtCO_2_, the remaining time the world can emit similar amounts of CO_2_ is only 11 years, with a 50% probability of remaining less than 1.5 °C warming.^17^

## Global Carbon Balance and Box Model

Since the beginning of the industrial revolution, humans have emitted an estimated 2,500 billion metric tons of carbon dioxide (GtCO_2_) from the combustion of coal, oil, and natural gas.^18^ Roughly one-quarter of that amount has been absorbed by the cool surface oceans at mid-to-high latitudes and transported down to the intermediate ocean (Figure 1).

Figure 1 is adapted and updated from a compartmentalized global carbon mass balance first published by Kwon and Schnoor in 1994.^19^ Mass transfer fluxes between compartments are shown on the arrows between boxes (GtC/year), and the “stocks” or total mass of carbon within each compartment are shown inside the boxes (GtC). Three large carbon stocks comprise the atmosphere, the terrestrial biosphere, and the ocean. The terrestrial biosphere is further compartmentalized into the land biota and soil and detritus boxes. The ocean is broken into surface oceans (100 m depth) and intermediate and deep ocean waters (3800 m). Surface oceans are divided into the cool surface ocean (1.21 × 10^14^ m^2^), where CO_2_ is more soluble, resulting in a substantial sink for atmospheric CO_2_, and the warm surface ocean (2.42 × 10^14^ m^2^), which is degassing ancient CO_2_ to the atmosphere from upwelling waters. The net mass moving by gas transfer from the atmosphere to the oceans is considerable, on the order of 3 GtC/year.

The speciation of carbon differs in various boxes. For example, carbon in the atmosphere is inorganic CO_2_, while carbon in land biota, soil/detritus, warm ocean biota, and cold ocean biota comprises total organic carbon (TOC). Warm ocean biota are all organisms from zooplankton to fishes in the warm surface ocean, whereas cold ocean biota are those residing in the cool surface ocean. TOC varies chemically and includes cellulosic plant biomass, woody biomass, roots, living organisms such as decomposers and bacteria, and even labile sugars, acids, and humic substances. Living organisms and detrital material include both particulate and soluble compounds in the Biota compartments of warm and cold ocean waters.

Plants comprise 82% of the biomass carbon in the terrestrial land biota (Figure 1), while bacteria, archaea, and fungi combined account for 16% of the total.^20^ Terrestrial animals (2 GtC) are only 0.4% of the total land biota, while marine biota (warm ocean biota and cold ocean biota) comprise 6 GtC, a 3 times larger store than land-based animals. Invertebrate mollusks, including those with calcium carbonate external shells, play an exceedingly important role in the marine food chain, yet they comprise only 0.2 GtC, while marine fish are a somewhat larger store at 0.7 GtC. Surprisingly, although humans affect all the carbon distributions on Earth, they constitute a paltry 0.11% of terrestrial carbon biomass (0.6 GtC out of 550 GtC).^20^ The mass of carbon in ocean waters is primarily composed of total inorganic carbon (TIC), with the dominant species as bicarbonate anions (HCO_3_^–^) (eq 4). Mass transfer into oceans of human-emitted CO_2_ is sufficient to have disrupted the chemical equilibria of the 1800s from about pH 8.2 to the present day of pH 8.05. Furthermore, due to the logarithmic scale of pH (−log{H^+^}), this slight decrease in pH due to human emissions have caused an increase in hydrogen ion activity {H^+^} of 40%! By far, the largest carbon stock in the oceans is TIC, located in intermediate and deep ocean waters, 38,000 GtC. It represents a compartment that exchanges over geologic time scales with the ocean bottom through volcanism, deep sea hydrothermal vents, and subduction; and with surface waters through vertical mixing, upwelling, chemical weathering, and precipitation.
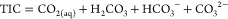
4

With current emission scenarios and projected increases in CO_2_, the pH of the ocean is projected to decrease to about 7.8 by 2100, causing it to become 140% more H^+^-acidic. This drop in pH will drastically affect coral reefs and animals that form calcium carbonate shells and skeletons, the base of the entire marine food chain.^21^

## Greenhouse Gases and Their Emissions Trends

**Figure 2:**
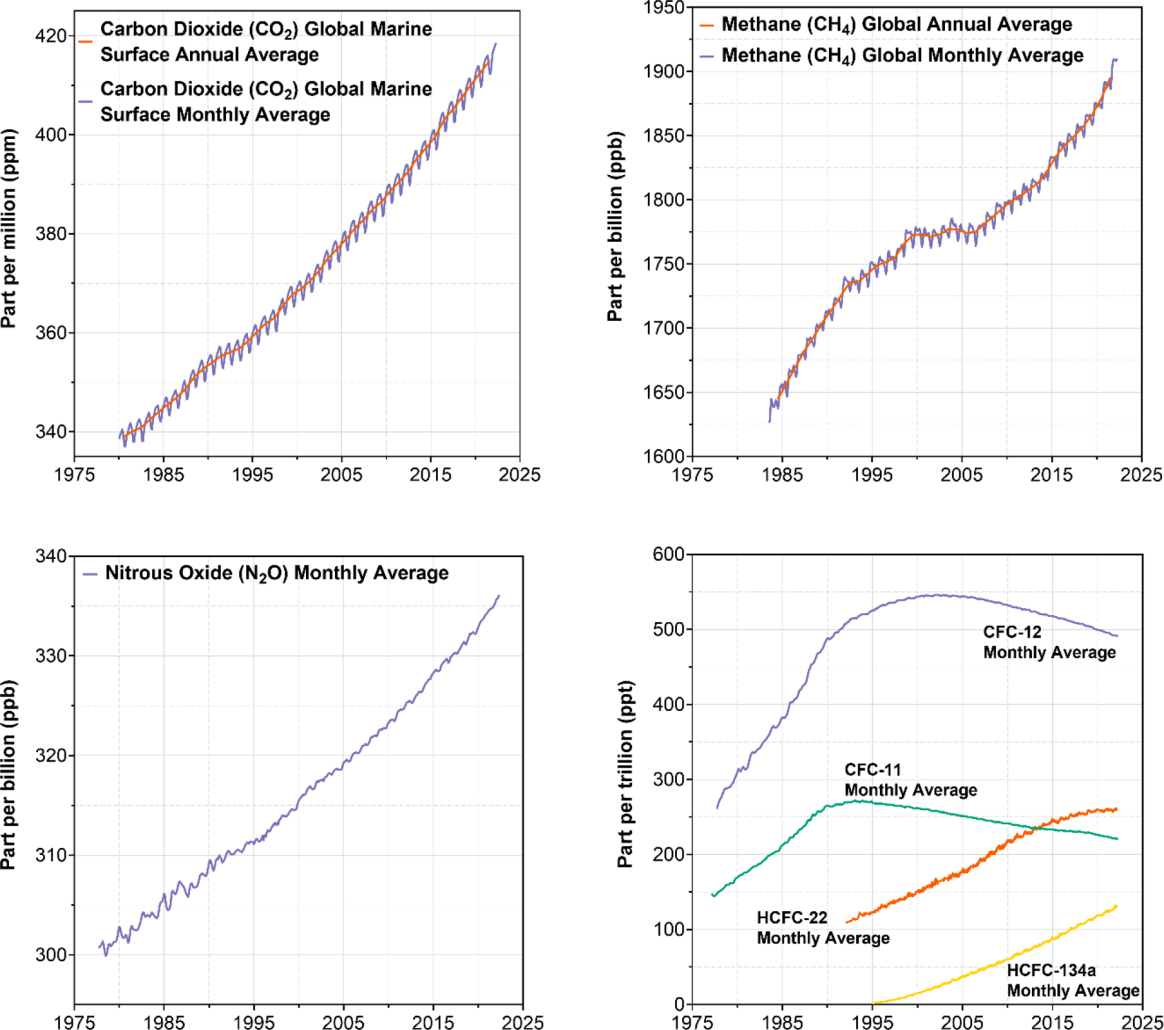
Trends in concentrations of major, well-mixed greenhouse gases: carbon dioxide, nitrous oxide, methane, and dichlorofluoromethane (CFC-12), trichlorofluoromethane (CFC-11), chlorodifluoromethane (HCFC-22), and 1,1,1,2-tetrafluoroethane (HCFC-134a). Original data obtained from NOAA Global Monitoring Laboratory.^22^

Greenhouse gases are long-lived in the atmosphere (long residence times), and thus, they can exert their heat-trapping effects for many years (Table 1). Figure 2 shows data from the National Oceanic and Atmospheric Administration (NOAA) on the most important greenhouse gases, CO_2_, methane (CH_4_), nitrous oxide (N_2_O), chlorofluorocarbons (CFCs), and hydrofluorocarbons (HFCs), which exert about 96% of the anthropogenic greenhouse effect.^22^ That heat-trapping effect is generated by the kinetic energy of these gases when they absorb infrared back-radiation. Gases with three or more atoms absorb energy at infrared wavelengths by vibrating and rotating. Carbon dioxide, methane, and nitrous oxide are all increasing due to fossil fuel combustion. Also, methane is emitted from various sources of natural gas, and N_2_O is released from increased fertilizer usage. In addition, increasing human population, consumption per capita, and burgeoning industrial/commercial activity (GWP) drive the increase in emissions of GHGs.

Carbon dioxide in Figure 2 is steadily increasing due to the continual burning of fossil fuels since the industrial revolution (eqs 1–3). As a result, small annual sinusoids are superimposed on the exponential trend, sometimes called the “lungs of the Earth.” Charles (David) Keeling was the first to observe the trend at Mauna Loa, Hawaii station. He began with funding for the International Geophysical Year in 1958, and the “Keeling curve” represents one of the most important geophysical records ever produced.^23^ Plants take up CO_2_ from the atmosphere in the spring when leaves grow and primary production increases. Subsequently, CO_2_ increases in the fall and winter when respiration returns CO_2_ to the atmosphere. Most of Earth’s land mass is in the northern hemisphere, so this effect is most pronounced in the north, and the two hemispheres are out of phase with respect to the annual sinusoidal increments superimposed on the overall increasing trend.

Thankfully, a “natural greenhouse effect” is responsible for keeping the Earth habitable. For example, water vapor (H_2_O) can also be considered a greenhouse gas and part of the natural greenhouse effect. This is because it has three atoms per molecule, absorbs infrared radiation at certain wavelengths, and vibrates when exposed, creating kinetic energy (heat). In fact, most of the natural greenhouse effect is due to the concentration of water vapor in the atmosphere (10–50,000 ppm), which causes the average temperature on the Earth to be 15 °C (59 °F) instead of −18 °C (0 °F). The second-most important gas contributing to the natural greenhouse effect is the steady-state balance of CO_2_ (280 ppm) that existed before the industrial revolution. Therefore, although anthropogenic GHGs have “forced” the warming of the Earth while raising humidity and water vapor in the air, H_2_O is not considered an “anthropogenic forcing” greenhouse gas. Instead, it is considered a “feedback effect” because it is not directly emitted by human activities, although it does contribute to overall global warming.

Methane is increasing in the atmosphere and is responsible for 17% of the anthropogenic greenhouse effect. Uncertainty surrounds the exact cause of methane increases. Possible sources include increased fracking and fossil fuel mining (coal, oil, and gas), increased ruminant animals for meat consumption, increased flooded rice agriculture for food, and increased leakage from permafrost during the thawing of Arctic lands.^21^

Human sources of reactive nitrogen far exceed natural sources from legumes and biogeochemical cycles. Perhaps 70% of increased nitrous oxide emissions (shown in Figure 2) result from nitrification and denitrification of fertilizers in agricultural soils. Global N-fertilizer applications have increased rapidly from 10 teragrams N per year (TgN/year) in 1960 to 110 TgN/year in 2017.^24^ Nitrogen fertilizers include urea, ammonia, ammonia sulfate, and ammonium nitrate. Eight billion people on Earth require more food and feed to be produced on (roughly) a fixed area of arable land to sustain increased crop yields.

Chlorofluorocarbons CFC-12 (CCl_2_F_2_) and CFC-11 (CCl_3_F) are refrigerants and blowing agents banned in the Montreal Protocol of 1987 (Figure 2). 197 countries ratified the Montreal Protocol, the first treaty in the United Nations’ history to achieve universal ratification. It is considered to be the most successful environmental agreement ever. The Protocol was necessary to reduce extremely stable ozone-depleting substances (ODS) capable of wafting all the way up to the stratosphere and destroying the ozone layer, which shields us from ultraviolet radiation. In the stratosphere, CFCs react with UV radiation to release chlorine atoms which rapidly destroy ozone, O_3_. Atmospheric lifetimes of CFC-12 and CFC-11 are 100 and 52 years, respectively, so it has taken a long time after the ban to observe a decrease in their concentration.^25^ Second-generation replacement chemicals HCFC-22 and HFC-134a (Table 1) with lifetimes on the order of 15 years have since been banned under the Protocol, but emissions are still substantial, so we have yet to see the decline in their atmospheric concentrations. Nevertheless, we can be grateful that the global community has taken action against the use of ODS chemicals because they are also highly potent greenhouse gases, which would by now have resulted in far more global warming than that of CO_2_.

## Temperature Change of the Atmosphere and Sea

**Figure 3:**
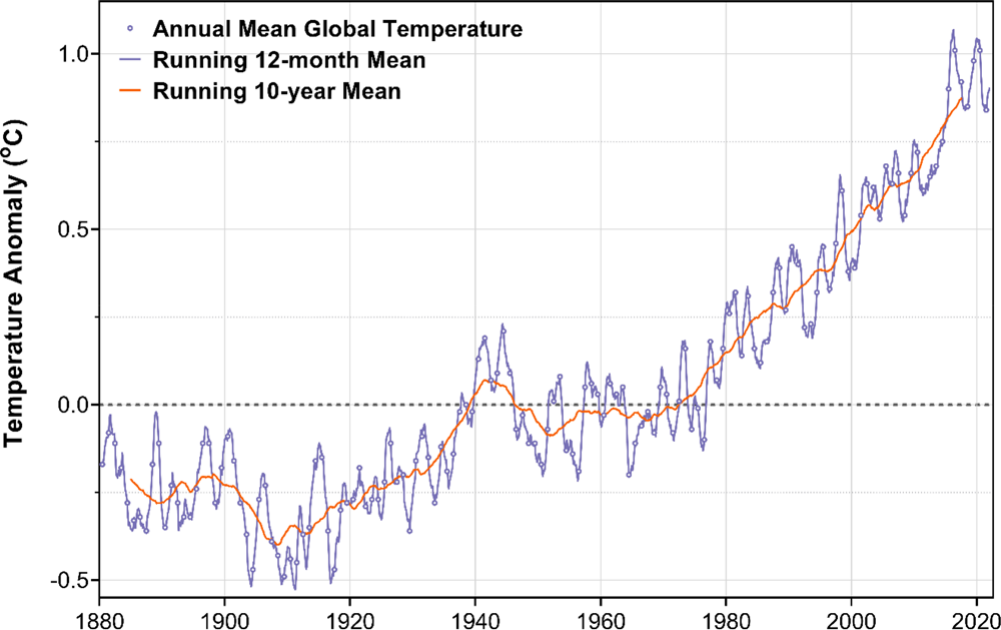
Change in global mean surface temperatures (1880–2022). Original data obtained from NASA GISS.^51^

When GHG emissions continue unabated, we expect an increase in global average temperature. Indeed, we have observed such an increase. The temperature anomaly (the change from a relatively stable temperature period of 1880–1920) is depicted in Figure 3 from Hansen, Sato, and Ruedy data.^26^ Increasing temperatures have been especially pronounced since 1970 when the signal clearly emerged from the interannual “noise”. The 1980s were warmer than previous decades, the 1990s warmer still, and so on for each decade following. The past eight years have been the eight warmest years in the instrumental record.

Super El Niños in 2015 and 2019 contributed to the warmest years on record, and we are currently experiencing a long La Niña in 2022–2023. It has caused relatively cooler global temperatures and extended drought and flooding in different regions. The net energy imbalance on Earth is approximately 1 W/m^2^—the power required to account for the warming of the planet over the entire year.^26^ Net anthropogenic GHG forcings are more than 3 W/m^2^,^21^ but the increased feedback of infrared radiation into space due to a warmer planet causes the net energy warming to be about 1 W/m^2^. Because of the enormous heat capacity of the oceans, most excess heat from our energy imbalance (more than 90%) accumulates in the oceans. According to data from the National Oceanic and Atmospheric Administration (NOAA), oceans are warming on the order of 1.5 × 10^22^ J/year, from the surface to a depth of 2000 m.^27^ By combining land and sea temperature databases, evidence indicates that the land is warming faster than the ocean but has less mass and heat capacity. As a result, ocean temperature increases lag the atmospheric warming, as is reasonable, given that excess heat is being transferred from the atmosphere downward into the oceans (top-down warming). The total land-based anomaly is now 1.1 °C (2.0 °F) and the average ocean sea surface temperature anomaly is about 0.9 °C (1.6 °F).

**Figure 4:**
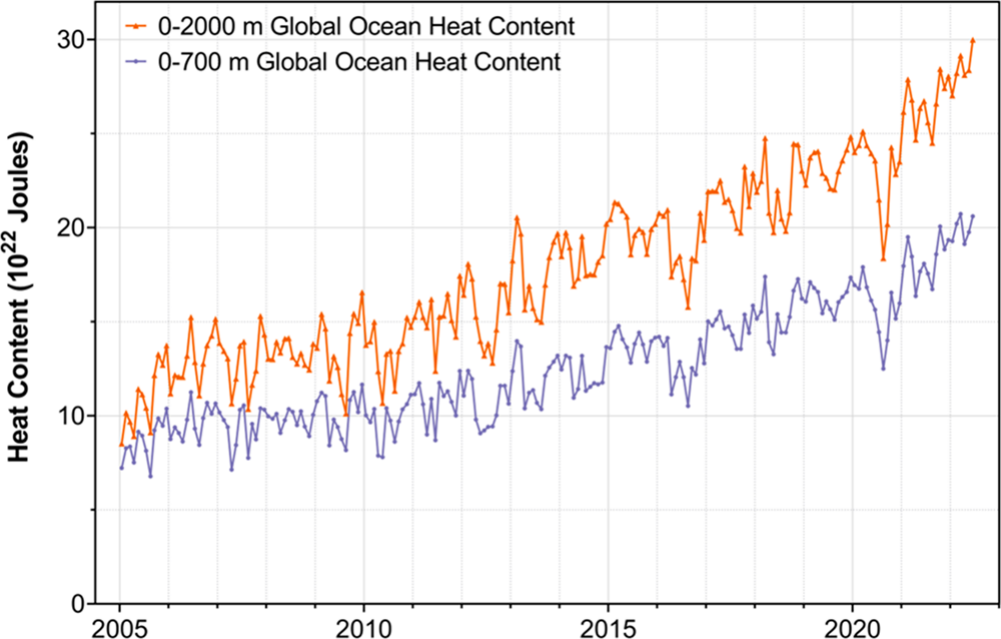
Global heat content of the ocean as measured by the Argo program. Original data obtained from NOAA/NESDIS/NCEI Ocean Climate Laboratory (2022).^52^

Figure 4 shows the heat added to the ocean, calculated from 4000 solar-powered floats in the ARGO international program. These floats undergo programmed dives to 2000 m while measuring temperature, pressure, and salinity very accurately—they transmit the data back to oceanographers who construct key results like Figure 4.^27^

Lakes can be viewed as an early warning system of future climate change in the ocean and coastal waters. O’Reilly et al. used 25 years of satellite and ground measurements for 235 lakes on six continents to show that lakes are warming much faster than the atmosphere or sea, at an average of 0.61 °F per decade (0.34 °C per decade).^28^ Increasing temperatures make lakes susceptible to harmful algal blooms that are toxic to fish and animals, low dissolved oxygen concentrations, and methane emissions.

## Physical Chemistry and the Master Variables of the Sea (*T*, pH (H^+^), pε)

Lars Gunnar Sillén is credited with emphasizing “master variables” in freshwater and marine chemistry.^29^ In his view, the master variables are those which allow acid–base reactions and oxidation–reduction reactions to be linearized in log–log plots. Thus, pH becomes a master variable for acid–base reactions, and oxidation–reduction reactions are linearized by pε = −log{e^–^}. Absolute temperature (actually inverse temperature) is the logical master variable in Arrhenius plots of reaction rate constants and Van’t Hoff plots for chemical equilibrium constants. Hence, the master variables: *T*, pH (H^+^), pε. Master variables control all the state variables of activities and concentrations.

Emissions of CO_2_ and other greenhouse gases have changed the Earth’s energy balance and raised the atmosphere’s temperature, which has, in turn, been transferred to the sea in a top-down fashion. We can observe the signal of the heat transfer down 2000 m into the ocean in a classical thermal-diffusion pattern. So, global warming has increased the sea surface temperature, the ocean’s heat content to a depth of more than 2000 m, the CO_2_ concentration, and H^+^ concentration in just a couple of centuries, with the largest changes occurring in the past 50 years. Given the enormous mass of the oceans (1.4 × 10^18^ Gt) compared to the atmosphere (5.25 × 10^15^ Gt), this is a formidable change in steady state and a serious challenge that we have created.

**Figure 5:**
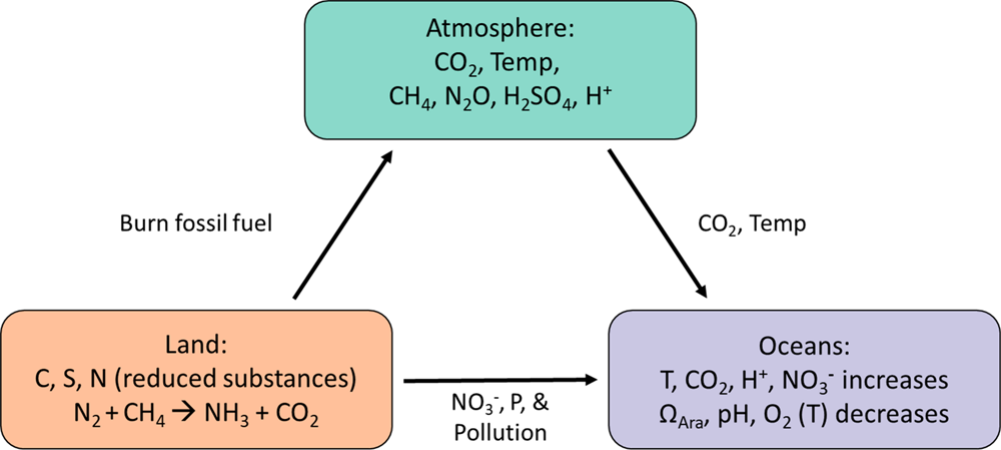
Schematic of the overall change in steady state and master variables due to massive disruption in elemental cycles on Earth by burning fossil fuels, global warming, and eutrophication of the oceans (Ω_ara_ = {Ca^2+^}{CO_3_^2–^}/*K*_so_ for aragonite, the calcium carbonate mineral in coral).

Figure 5 is a schematic of the overall changes in steady state caused by the disruption of land, atmosphere, and oceans. We see that master variables in the physics and chemistry of the sea have been altered, including the temperature, pH, and pε (oxidation–reduction potential). Warming causes glaciers to melt, ice sheets to collapse, and a freshening of the sea’s salinity near glaciers and ice sheets. In turn, warming alters density stratification, vertical mixing, the spin-up of hurricanes, and large-scale ocean circulation.

Humans are rivaling nature’s elemental mass and differential energy flows. Already, climate change is widely accepted to have passed acceptable planetary boundaries. In addition, vast applications of nitrogen (and phosphorus) fertilizers have likely transcended planetary boundaries.^30,31^ Human activities surpass natural N cycles, add greenhouse gas concentrations at unassimilable rates to the atmosphere, and discharge fertilizers and xenobiotic substances into the sea.

Pollution of the sea includes persistent, bioaccumulating, and toxic chemicals (PBTs) such as PCBs, PAHs, PFAS, mercury, and micro- and macroplastics. Some effects of these substances are triggered or exacerbated by temperature. We are likely to exceed the goal to limit climate change to within the UN Framework Convention on Climate Change (UNFCCC) and Paris Agreement to “well within 2.0 °C” warming (preferably 1.5 °C).^26^ Unfortunately, it appears that we will surpass 1.5 °C warming within the next 20 years at current rates of 0.18 °C/decade (Figure 3). GHG emissions must plummet by 50% by 2030 and to “net zero” by 2050 to slow the current rate of warming and limit warming to 1.5 °C. Let us examine the overall implications of these changes for a steady-state sea.

## *P*_CO_2__ and pH of the Sea

**Figure 6:**
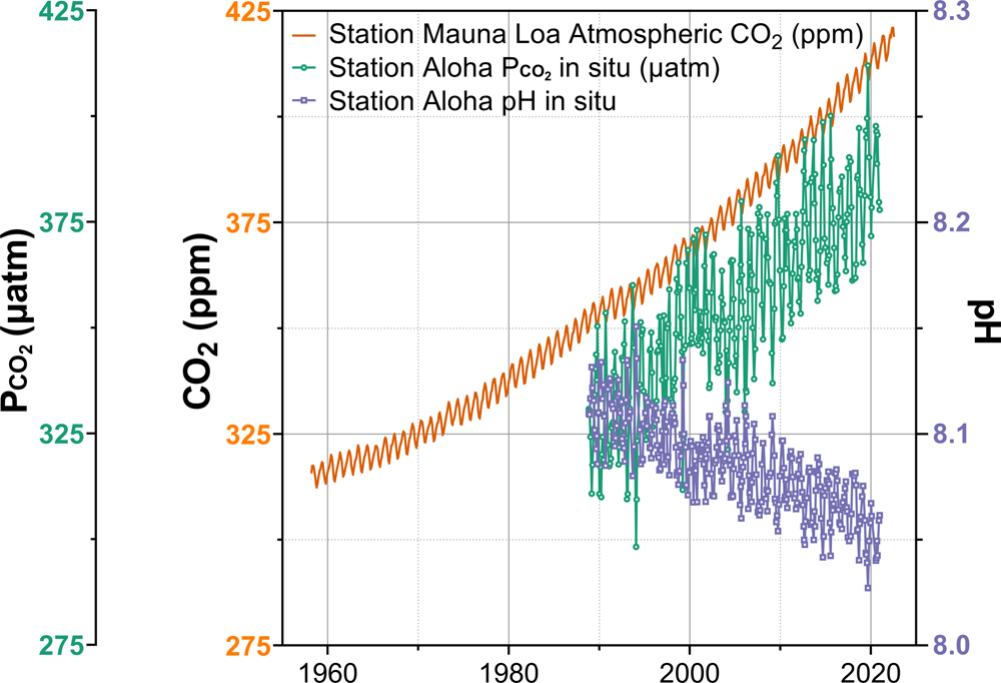
*P*_CO_2__, CO_2_ concentration, and pH of the North Pacific Ocean. Original data obtained from Dore et al., 2009, NOAA ESRL, and PMEL Carbon Program.^12,52,53^

Figure 6 shows the increasing atmospheric CO_2_ concentration and the corresponding increase in partial pressure of CO_2_ (*P*_CO_2__) in seawater, demonstrating linearity and the principle of gas exchange and solubility (Henry’s Law) at the sea surface. [H_2_CO_3_*] in seawater is the sum of dissolved CO_2(aq)_ plus true molecular [H_2_CO_3(aq)_] as described in eq 5 below. True molecular [H_2_CO_3(aq)_] is very small relative to [CO_2(aq)_], which predominates. Henry’s Law applies (eq 6), and [H_2_CO_3_*] undergoes an acid dissociation *K*_a1_ to form hydrogen ions and bicarbonate.^32^ Furthermore, bicarbonate ions are amphoteric—they can further dissociate to form hydrogen ions plus carbonate anions (eqs 7–10). Thus, the disruption of steady state in the atmosphere for the partial pressure of CO_2_ transfers to a change in master variable pH in the sea and concentrations of [CO_2(aq)_], [H^+^], and TIC. Alkalinity (also known as acid-neutralizing capacity, ANC) in eq 11 does not change because the increase in [HCO_3_^–^] exactly offsets the increase in [H^+^] when [H_2_CO_3_*] dissociates (eq 7).
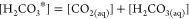
5
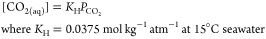
6
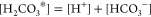
7
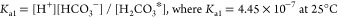
8
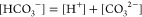
9
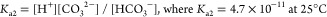
10
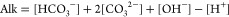
11As sea surface temperatures increase, the equilibrium constants in eqs 8 and 10 increase, causing [H_2_CO_3_*] to produce more [H^+^] and [HCO_3_^–^] ions.

Seawater comprises dissolved neutral salts of weathering products with 2 mequiv/L of bicarbonate alkalinity buffering included. The ionic strength of open ocean water is approximately I = 0.7 M, and it consists primarily of NaCl_(aq)_ with some MgSO_4_ and CaSO_4_ added, such that any change in the weak acid [CO_2(aq)_] causes a significant decline of seawater pH through eq 7. Alkalinity can also be expressed by a charge balance. It is the sum of the base cations minus the sum of the acid anions, as depicted in eq 12 below.
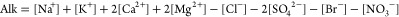
12

The net effect of increasing [CO_2(aq)_] is to reduce carbonate ion concentrations [CO_3_^2–^] in the ocean (eq 13), which directly impedes the precipitation of calcium carbonate for skeletal coral reefs and the formation of shells for pteropods at the base of the marine food chain.
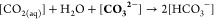
13

Nitrate concentrations in surface seawater vary from nearly zero in the tropics and subtropics to 50 μM in cold temperate and Arctic and Antarctic Oceans. Any change in nitrate concentrations due to fertilizer runoff from land can enrich the primary productivity of coastal waters and, eventually, the open sea. Furthermore, enhanced algal productivity in the open ocean will eventually cause subsequent declines in dissolved oxygen as algal biomass cells respire and are biodegraded (the respiration back-reaction in reaction 9 of Table 2).

**Table 2:** Some Processes That Modify Alkalinity and Dissolved O_2_ Concentrations in Ocean Waters When the Reaction Proceeds from Left to Right

reaction	change in alkalinity or [H^+^] consumed	change in oxygen or [O_2_] produced
	(equivalents consumed or produced)	
(1) dissolution of calcite and aragonite	+2	0

(2) ionization of CO_2(aq)_ as a weak acid	–1	0

(3) boric acid as a weak acid	–1	0

(4) oxygen depletion by respiration and biodegradation	0	–1

(5) nitrogen fixation by autotrophic diazotrophs	+2	+3/2

(6) nitrogen fixation by heterotrophic diazotrophs	+2	+1/2

(7) nitrification in aerobic portions of ocean water	–1	–2

(8) denitrification in deeper anoxic ocean water	+1	0

(9) algal biomass photosynthesis (production and respiration)	+18	+138


Oxidation reactions cause alkalinity to decrease, such as the nitrification reaction 7, Table 2. Oxidation of dissolved organic matter, represented by the carbohydrate (CH_2_O) in reaction 4 of Table 2, is also acidifying because it produces [CO_2(aq)_] that decreases pH. However, alkalinity is unchanged by CO_2_ production. Reduction reactions cause alkalinity to increase, as shown by reactions 5, 6, and 8 in Table 2. If algae utilize [NO_3_^–^] as opposed to [NH_4_^+^] as their nitrogen source, the photosynthesis process (reaction 9 in Table 2) is alkalizing. A typical Redfield stoichiometric formula for algal biomass is shown, but it ignores trace elements such as reduced iron species [Fe(II)] that limit algal production in the high latitudes of the South Pacific and Arctic Oceans. Microbes and their enzymatic systems mediate all of the reactions 4–9 in Table 2.

Oxygen depletion of the open ocean is a serious problem and is represented by reaction 4, Table 2, and the reverse reaction of photosynthesis, reaction 9. While nitrification (reaction 7) depletes dissolved oxygen concentrations, it is a relatively minor sink. Nitrogen fixation by diazotrophs in ocean water is a significant process that generates reactive nitrogen species for primary productivity in the open ocean, and it serves to add oxygen, as shown by reactions 5 and 6 in Table 2. Photosynthesis is the primary process for the addition of dissolved oxygen in the euphotic zone, the upper 200 m of ocean water (reaction 9 in Table 2). Utilizing the sun’s energy in photosynthesis drives the master variable pε, that is, the oxidation–reduction potential of the entire sea.

Thus, fossil fuel emissions cause two major problems for Earth. First, the obvious problem is the warming of air, land, and sea temperatures and the consequent extreme events that result from the planet processing more energy, resulting in floods, droughts, wildfires, and sea level rise (storm surge). The second problem is often unrecognized or underappreciated by the public. Carbon dioxide acidifies the ocean, and the change in steady state from pH 8.2 to pH 8.05 today is causing difficulty for marine organisms to form calcium carbonate shells and skeletons. Oceans will continue to acidify in the future due to CO_2_ emissions, jeopardizing the entire marine food chain, unless fossil fuel burning ceases, and then Earth could begin to return to its original steady state. Before the industrial revolution, the steady-state concentration of CO_2_ was 280 ppm, but it is now approaching 420 ppm, an increase of 50% and accelerating. The pH of the sea has not been as acidic as projected for the year 2100 (pH 7.8) in hundreds of thousands of years, and it will take a couple of hundred years to restore the oceans to preindustrial revolution levels after we curtail CO_2_ emissions.^22^

## Aragonite and Calcite Precipitation and Saturation Indices

Increasing carbon dioxide from the atmosphere adds TIC to ocean water, and the net effect reduces carbonate concentrations and shifts the chemical equilibrium to bicarbonate ions at a lower pH (eq 13). Bicarbonate and carbonate anions are the principal buffer in ocean water and provide some measure of protection against acidification. In addition, carbonate anions are required for the precipitation of calcium carbonate, CaCO_3(s)_, following thermodynamics with a defining equilibrium constant *K*_so_, solubility product (eqs 14 and 15).
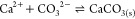
14
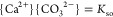
15

There are at least five crystalline forms of calcium carbonate in nature, but calcite and aragonite are the most important in oceans. Different crystal structures of the same chemical formula are termed “polymorphs.” Aragonite crystals are orthorhombic in architecture, and calcite crystals are trigonal. Calcite is the least soluble (most stable structure), and aragonite is somewhat more soluble (dissolves more easily). The *K*_so_ for calcite in seawater at 15 °C and salinity of 35,000 is 10^–6.3628^, and that for aragonite is slightly greater at 10^–6.1153^.^33^ Thus, aragonite is approximately 1.5 times more soluble than the calcite polymorph.

Major types of phytoplankton, coccolithophorids, and foraminifera create calcite shells, while two taxa of zooplankton, pteropods, and heteropods, form shells of aragonite. Both tropical and cold-water corals are formed from aragonite, and aragonite is usually predominant in seston surveys as particles fall through the photic zone (200 m) of ocean water. Usually, seston particles become dissolved (their organic carbon is oxidized), and their nutrients are solubilized to be mixed and returned to the surface ocean once again.

**Figure 7:**
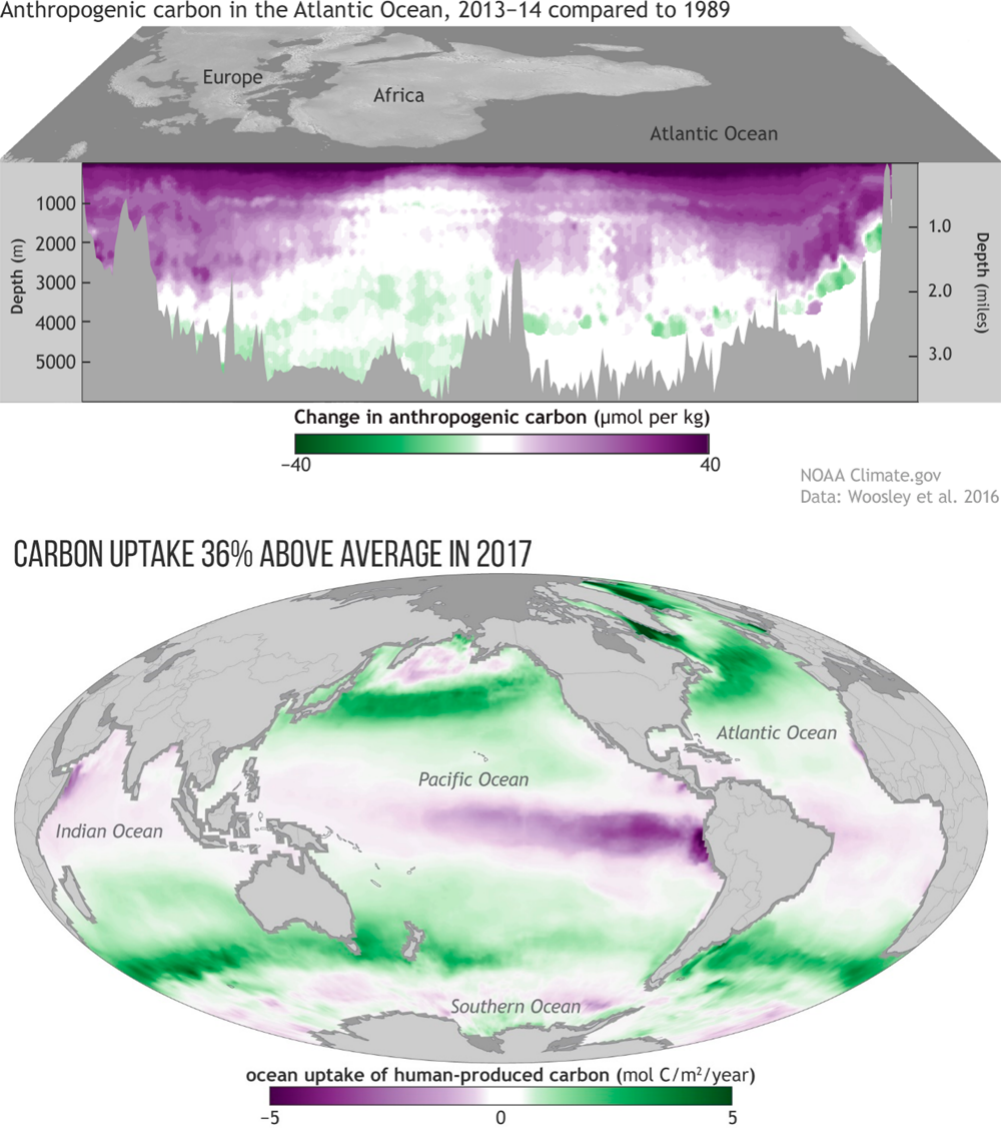
(Top) Change in anthropogenic carbon in the Atlantic Ocean, 2013–2014, compared to 1989. (Bottom) Oceanic uptake of anthropogenic carbon, 2017. Reprinted with permission from (1) Sullivan, C. L., Rebecca;. *2017 State of the Climate: Ocean Uptake of Human-Produced Carbon*. NOAA Climate.gov, 2018; https://www.climate.gov/news-features/featured-images/2017-state-climate-ocean-uptake-human-produced-carbon (accessed 2022 October).^54,55^ Not subject to U.S. Copyright.

Simultaneous heat and mass transfer result in CO_2_ being transferred into the cold water oceans where CO_2_ has greater solubility (Henry’s Law constant, *K*_H_, temperature dependence), as illustrated in Figure 7. Warm equatorial surface ocean waters are degassing CO_2_ from upwelling waters, but these molecules are composed of CO_2_ absorbed by the sea centuries or millennia ago. The net effect on the sea is the uptake of CO_2_ and a concomitant decrease in average surface pH. The top panel of Figure 7 shows that human-emitted CO_2_ concentrations in the cold oceans of the North and South Atlantic Oceans have penetrated as deep as 3000 m since the baseline year of 1989. As global warming progresses, less CO_2(g)_ will transfer from the atmosphere to the ocean surface as predicted by the Van’t Hoff relationship and decline in *K*_H_, eq 6. This, in turn, will cause the ocean sink (net uptake by the ocean) to slow, and a larger fraction of CO_2_ will remain in the atmosphere as the CO_2_ concentration increases.

While aragonite is more soluble than calcite, it is the polymorph most frequently precipitated by ocean organisms to make coral. Thus, the solubility of aragonite is of prime importance to the base of the food web and the health of the sea. The ion activity product, IAP = {Ca^2+^}{CO_3_^2–^}, shown in eq 14, defines the conditions of aragonite saturation. Ocean water is considered saturated with aragonite when the ion activity product is equal to the *K*_so_ for aragonite. When the ion activity product is less than the *K*_so_ for aragonite, ocean water is considered to be under-saturated, in which case aragonite shells would dissolve over time, and animals would expire. However, only in scientific experiments have conditions been so severely under-saturated thus far. When the IAP is greater than the K_so_ for aragonite, the solution is supersaturated, which is currently the case. It means that aragonite precipitates spontaneously according to the laws of thermodynamics at chemical equilibrium. Spontaneous precipitation of aragonite makes it easier to form coral and the shells of pteropods and heteropods. The Greek symbol for omega, Ω, with a subscript ara, is used to denote the aragonite saturation, i.e., the ratio of IAP/K_so_ for aragonite. The ocean at tropical and subtropical latitudes, where most coral is found, tends to have Ω_ara_ of 3.0–4.0 (an IAP that is 3 or 4 times the *K*_so_ value). Corals in northeastern Australia, the Yucatan, and Baja Mexico are currently around 3.0, already marginal to poor for coral reef growth.^34^

Conditions for coral reef growth are as follows:

- Ω_ara_ > 4.0: optimal
- 3.5 < Ω_ara_ < 4.0: adequate
- 3.0 < Ω_ara_ < 3.5: marginal
- Ω_ara_ < 3.0: poor

**Figure 8:**
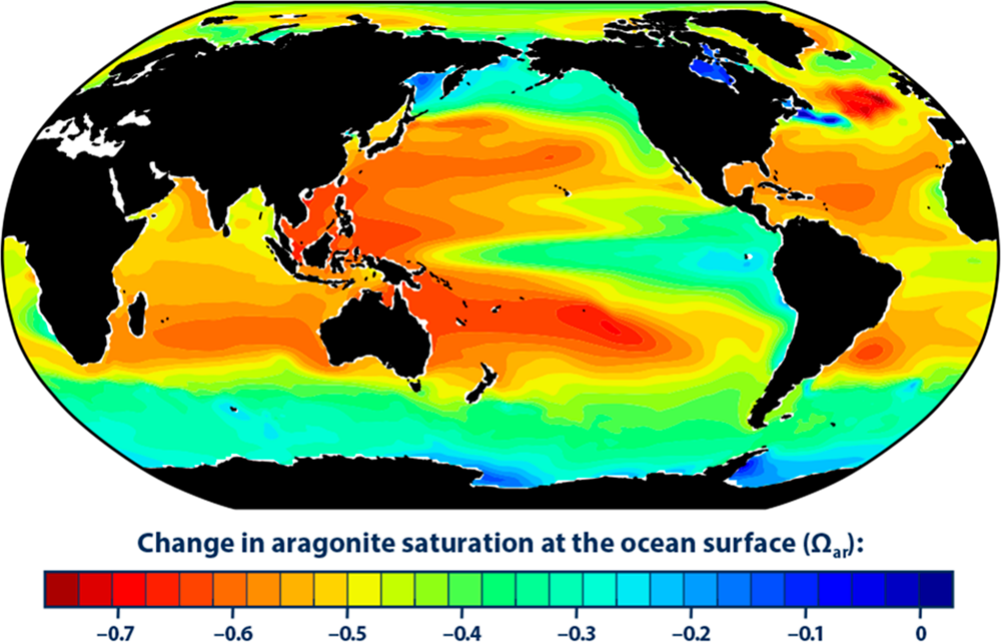
Aragonite and calcite saturation indices; change in aragonite saturation, 1880–2015. Reprinted with permission from U.S. Environmental Protection Agency. *Climate Change Indicators in the United States. [Change in Aragonite Saturation]*, 2016; https://www.epa.gov/climate-indicators/climate-change-indicators-ocean-acidity#ref11 (accessed 2022 September).^56^ Not subject to U.S. Copyright.

Most concerning is that the carbonate concentrations of the sea have been declining steadily as CO_2(aq)_ increases with climate change and the pH drops. Figure 8 shows a decline of 0.5–0.6 units^35^ in the period from 1880 to 2015 for most of the locations where coral reefs grow, including the Great Barrier Reef in northeast Australia, the South Pacific and western Pacific islands area, Baja California, the Caribbean, and the Gulf of Mexico. Australia, South Asia, Yucatan, Baja Mexico, and the east coast of Central America seem particularly vulnerable to coral reef decline with aragonite saturation losses of 0.5–0.6 and Ω_ara_ of 3.0 or less.^34,35^ A looming question is whether some coral reefs can acclimate and adapt to declining pH and Ω_ara_ values as climate change continues. A recent study of reefs near the Palau archipelago in the South Pacific has shown some tendency of local coral to adapt to Ω_ara_ values as low as 2.44.^36^

Still another master variable, temperature, plays a role in coral reef decline. A symbiotic relationship exists between coral and tiny algae (zooxanthellae) living inside their polyps. Coral feeds on the photosynthetic products of algae, and algae are provided a protected habitat and nutrients from the coral. As ocean temperatures increase (or pollution inundates the coral), algae tend to depart coral tissue, causing it to lose its beautiful colors and become bleached. Without food from algae, the coral may succumb to disease and death.

## Haber-Bosch, Nitrate, and Nutrient Pollution

The Haber-Bosch process was developed in the early 1900s for converting nonreactive nitrogen gas into ammonia for fertilizers and other uses. It completely transformed the nitrogen balance on Earth. As shown by eq 16, hydrogen atoms are abstracted from natural gas (methane) in the presence of catalysts and under high temperature and pressure to convert nonreactive N_2(g)_ to reactive ammonia.^10^ Not only does the process account for a huge amount of man-made reactive nitrogen in the form of fertilizer ammonia, but it also consumes a significant quantity of natural gas and emits carbon dioxide GHGs.
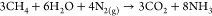
16

Reactive nitrogen (N_r_) is biologically, photochemically, or radiatively active (NH_3_, NH_4_^+^, NO_2_^–^, NO_3_^–^, NO, N_2_O) in contrast to N_2(g)_, which comprises 79% of the atmosphere but is relatively inert. Today, human-produced reactive forms of nitrogen via the Haber-Bosch process, fossil fuel combustion, and legume agriculture far exceed Earth’s natural sources sequestered by biological nitrogen fixation (BNF).^37^ According to Galloway et al., humans produce roughly twice as much reactive nitrogen (∼210 Tg N/year) relative to all-natural BNF sources.^38^ Natural BNF sources use the nitrogenase enzyme system and utilize nonreactive nitrogen gas to produce NH_3_ (eq 17). In BNF, prokaryotes (single-cell bacteria and cyanobacteria called diazotrophs) are primarily responsible for the fixation of reactive nitrogen in the ocean and keeping the nitrogen cycle spinning. Photosynthetic marine prokaryotes are responsible for most of the nitrogen fixation that drives biological productivity in the open ocean (106–120 Tg N/year), far from the continents and terrestrial N runoff.^38^ Land-based plants (legumes), both naturally occurring and as food crops, also contain the nitrogenase enzyme. They fix N_2(g)_ via rhizobia in root nodules. Nitrogenase enzyme systems function under anoxic conditions; i.e., they must be protected from oxygen. Thus, they are encased inside root nodules or as heterocysts in cyanobacteria.^37^
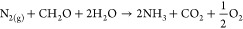
17

Denitrification is the major loss mechanism for reactive nitrogen in the oceans. It is microbially mediated by facultative and anaerobic bacteria deep in the water column and/or sediments. Nitrate is converted to nonreactive N_2_ with the enzymatic aid of denitrifying bacteria and a carbohydrate/energy source from dissolved organic matter, CH_2_O (eq 18).^10^
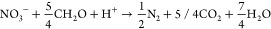
18

Primary production in the euphotic zone (upper 200 m) is responsible for the continuous production of algae (phytoplankton) in the sea (eq 19). Photosynthesis of algal biomass, C_106_H_263_O_110_N_16_P_1_, represents the forward reaction, while the back reaction reflects respiration and/or death and decay.^10^ We owe the phytoplankton stoichiometry in eq 19 and Table 2 to the seminal ocean research of Redfield.^39,40^ Excessive additions of nutrients can cause severe problems when the forward reaction of eq 19 becomes too great, and the subsequent respiration and death of the algal biomass results in the consumption of dissolved oxygen and sinking of dead particulate matter (seston). This reverse reaction of eq 19 represents the respiration and mineralization of dead algae and the return of nutrients to the water column. Seston particles also serve as a food source for heterotrophic bacteria, which consume dissolved oxygen by utilizing the carbon and energy source represented as CH_2_O in eq 20.
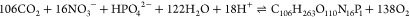
19
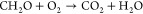
20

In shallow coastal areas near continents, this photosynthesis/decay/mineralization cycle is driven by reactive N runoff from agriculture, N deposition from polluted air, and domestic waste treatment discharges (cultural eutrophication). As a result, the overenrichment of coastal waters has become a severe problem, causing the chronic loss of dissolved oxygen and hypoxia (dead zones) along the coasts. Over 400 hypoxic areas were reported by Diaz and Rosenberg worldwide in 2008.^41^ Perhaps most concerning, deeper ocean waters are beginning to reflect low dissolved oxygen concentrations and excessive nutrient fluxes due to warmer ocean waters and pollution.

## Dissolved Oxygen and the Sea

Dissolved oxygen (DO) in the ocean is critical for biodiversity, marine ecosystems, coral reefs, and fisheries to survive. We are using DO synonymously with the chemical terminology, O_2(aq)_. When oxygen is consumed at rates greater than it is supplied, the ocean’s O_2(aq)_ content decreases, and both coastal areas and the open ocean are vulnerable. Oxygen is supplied by photosynthesis and gas transfer at the ocean’s surface by the partial pressure of oxygen in the atmosphere, 20.3% (0.203 atm at sea level and 20 °C). Oxygen is less soluble in warmer waters, while climate change causes sea surface temperatures to increase, reducing oxygen concentrations. Reductions in O_2_ solubility in warmer surface seas account for about 15% of the declining oxygen problem.^42^ However, the mass transfer of oxygen downward is also limited by stronger ocean thermal stratification, and together with increased microbial respiration due to increased temperature and nutrient enrichment, they account for an additional 85% of the loss of oxygen in deep ocean water.^42^ Brewer makes a strong point that the rising temperature of the sea has provided the driving force for increased microbial respiration (Arrhenius relationship), contributing to declining oxygen and that thermal change in the ocean is responsible for many deleterious ecological shifts like poleward fish migration.^43^

**Figure 9:**
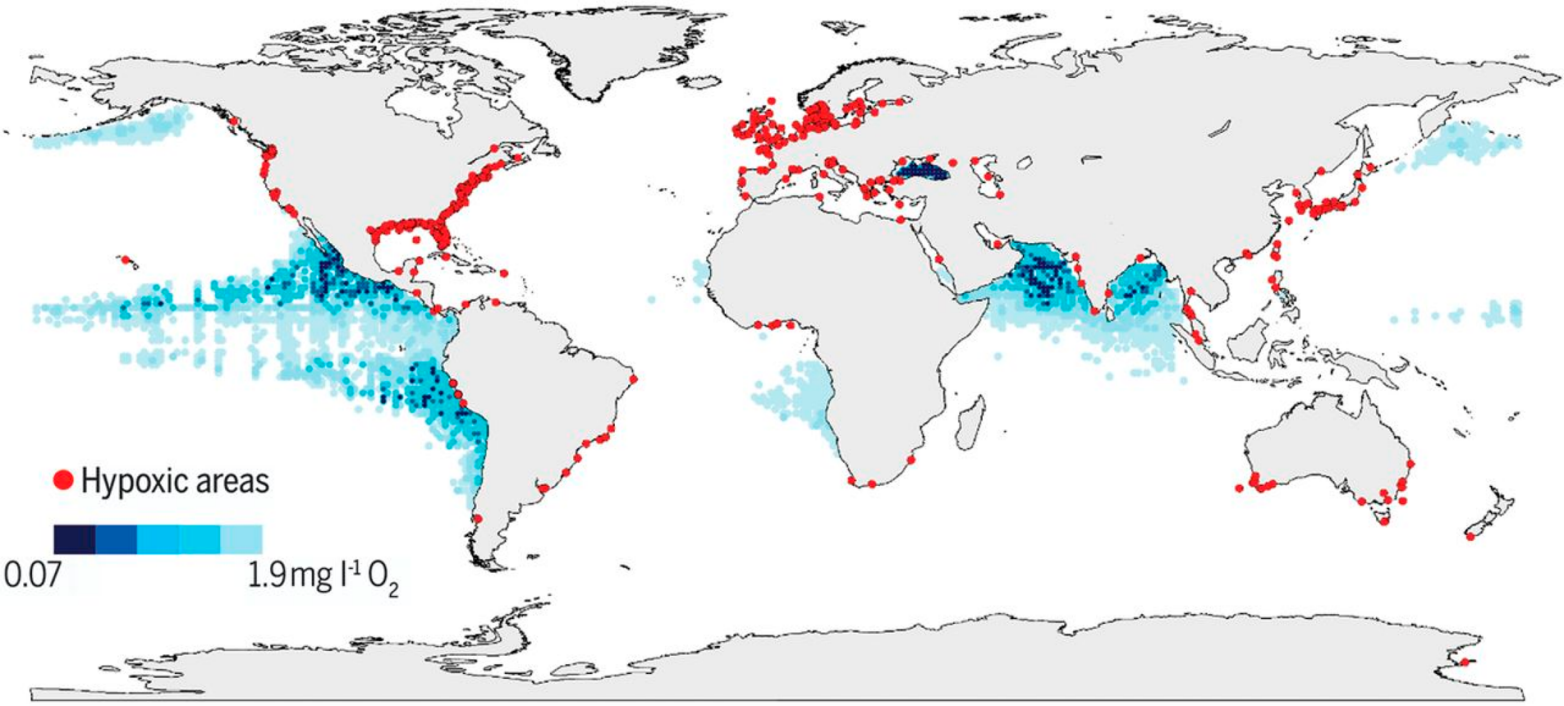
Ocean deoxygenation around the globe as of 2009. Red dots indicate areas of hypoxia (<2 mg/L, <63 μmol/L O_2_) in coastal waters due to anthropogenic pollution. Blue shaded areas indicate areas of high deoxygenation at 300 m depth. Reproduced with permission from ref (44). Copyright 2018 AAAS.

Figure 9 shows shaded areas of the ocean where oxygen is depleted by red dots delineating coastal hypoxia around the world.^44^ The open ocean concentrations of dissolved oxygen dip as low as 0.07–1.9 mg/L. Most aerobic organisms cannot survive under these low oxygen conditions. Only anaerobic or facultative bacteria, archaea, and some specialized deep water organisms can survive such low DO conditions. The UNEP Open Ocean Assessment has reported that sizable open ocean areas contain <100 μmol O_2_ per kg seawater at 200–600 m depth. Saturated conditions result in concentrations of 162 μmol O_2_ per kg seawater, about 9 mg/L.^45^

A major cause of low DO in the open ocean is that microbial respiration of dissolved organic carbon (accelerated by increased ocean temperatures and nutrient concentrations) has caused oxygen concentrations to decline at depths of 100–1000 m. Furthermore, this phenomenon, is exacerbated by increased thermal stratification, which slows downward oxygen transfer. For example, Schmidko et al. reported a 2% overall decline in the global oceanic water column from 1960 to 2010.^46^ They found that oxygen content declined by 4.8 ± 2.1 petamoles (10^15^ mol) from a total of 227.4 ± 1.1 petamoles in 1960. A two percent decline may not sound very harmful, but it represents 154 billion metric tonnes of oxygen and indicates a serious trend, especially for deep sea fisheries and oceanic ecosystems.

Coastal hypoxia (red dots in Figure 9) illustrates the connection between local/regional and global problems. Eventually, local hypoxic areas grow larger with continued nutrient and temperature inputs, becoming regional or even global (open ocean) problems. The time scale of transport between coastal waters and the open ocean is on the order of 10–100 years. Thus, the decline of DO at 300 m depth (Figure 9) and 200–600 m depth (UNEP Open Ocean Assessment) indicate that regional effects are indeed becoming of larger global scale.

Since the 1960s, low-oxygen areas in the open ocean have increased by 4.5 million km^2^, and over 500 low-oxygen sites have now been identified in estuaries and other coastal water bodies.^42^ The Baltic Sea is the largest site with DO concentrations less than 2 mg/L over 80,000 km^2^.^42^ Second largest dead zone is the Gulf of Mexico near Louisiana, which measured 17,000 km^2^ in 2021.^47^ The Black Sea has the largest volume of anoxic waters and sulfide-rich sediments.^48^ Human activities are a significant cause of oxygen decline in both the Open Ocean and coastal waters. In addition, inputs of reactive nitrogen compounds to the sea have more than doubled during industrialization over the past 150 years. Overfertilization and the resulting decline in Open Ocean DO now loom as serious threats to both coastal and pelagic ecosystems.

## pε of the Sea

What is the pε of the sea? The question was first asked and answered by the famous Swedish chemist Lars Gunnar Sillén as early as 1965.^29,49^ Werner Stumm, another giant in the field, elaborated on the point in 1978.^50^ The answer is 12.5. At least, that was the answer in the 1960s. As we have seen in this review, the open ocean is beginning to change its master variables of temperature, pH, and pε due to a massive disruption in elemental cycles that we call climate change, together with excessive nutrient additions from coastal waters. These forces upset the long-enduring pseudo-steady state of the Sea.

Like pH, pε is a master variable of chemistry that determines oxidation–reduction in aqueous systems. It is defined as the negative logarithm of electron activity. Of course, naked electrons do not actually exist, just as free protons H^+^ do not exist either, but it is a convenient construct to consider a hypothetical electron activity, pε.
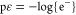
21

In the ocean, equilibrium redox chemistry is composed of a number of redox couples of known concentration and activity. For example, redox couples include O_2_/H_2_O, NO_3_^–^/N_2_, CO_2_/CH_4_, MnO_2_/Mn^2+^, NO_3_^–^/NH_4_^+^, FeOOH/FeCO_3_, SO_4_^2–^/HS^–^, CO_2_/CH_4_, N_2_/NH_4_^+^, and CO_2_/CH_2_O. We know photosynthesis drives oxygen production at approximately 42 {e^–^}_eq_/m^2^·year,^50^ which dominates the euphotic zone and keeps the system at disequilibrium. Dissolved oxygen is a strong oxidant that should not coexist with organic matter and trace concentrations of highly reduced substances such as CH_4_, CO, and H_2_. At chemical equilibrium, all reduced substances should be fully oxidized by O_2(aq)_. Thus, the ocean cannot be truly characterized by a unique pε. However, the redox components, while not at chemical equilibria, are nearly in a steady state driven by the sun’s energy, and oxygen is dominant in regulating the redox properties of ocean water. Most other redox couples are very slow in reaching chemical equilibrium, and they do not couple (react) with one another readily. Therefore, the controlling half-reaction is the redox couple between O_2(aq)_ and H_2_O (eq 22) driven by photosynthesis.
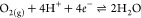
22The pε is defined by the Nernst equation and the oxidation intensity, *E*_H_, of the system in eq 23.
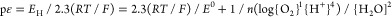
23The first term 2.3(*RT*/*F*)/*E*^0^ is the standard state of electron activity or pε^0^. Under the current ocean conditions at pH 8.1 and *P*_O_2__ of 0.203 atm, eq 23, yields a pε of 12.5 (eq 24).
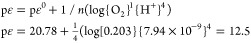
24

If oxygen concentrations decrease, as they have done in the shaded areas of Figure 9, to less than 1.9 mg/L, then the partial pressure of oxygen would decrease from 0.203 to about 0.0254 atm. That causes the pε of the deep ocean to decrease from 12.5 to 12.23, a significant decline indicating a more reducing condition where compounds like NH_4_^+^, Fe^2+^, CH_4_, HS^–^, and N_2_O could become more predominant, and ecosystems and fisheries could suffer, much as they do in coastal hypoxia.

## Summary and Prognosis

**Figure 10:**
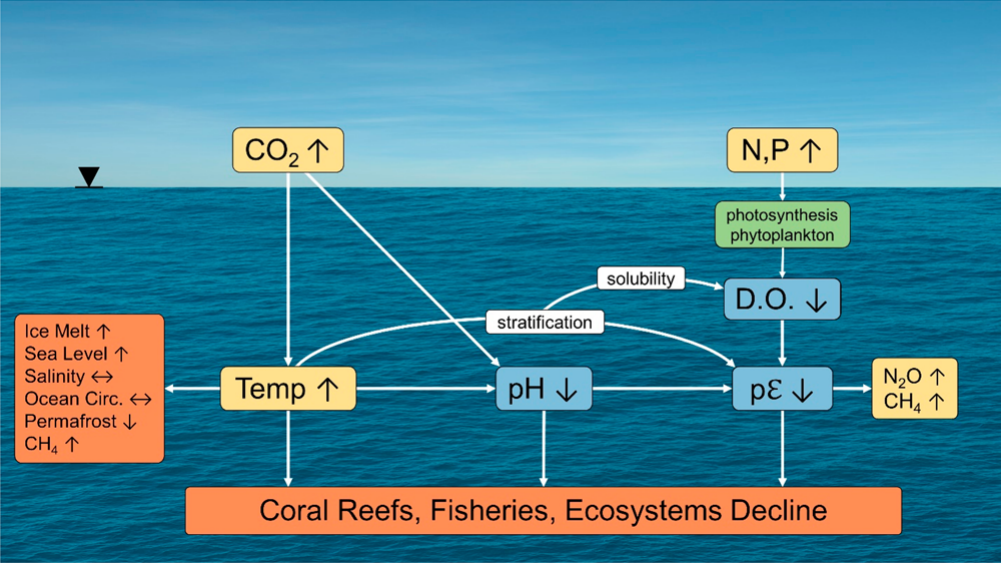
Anthropogenic activities impact the ocean’s master variables, temperature, pH, and pε. These master variables control the oceans’ physical, chemical, and biological processes, such as stratification, circulation, oxygen solubility, ice melt, sea level rise, and redox processes. In the end, our coral reefs, fisheries, and ecosystems are at risk.

A massive disruption in elemental cycles has upset the steady state and changed the master variables of the atmosphere, land, and ocean. The massive flux of C, N, and S, together with the overfertilization of coastal zones with N and P, have disturbed the energy balance of Earth and the pseudo-steady state of atmosphere and ocean, which in turn has changed the master variables of temperature, pH, and pε in the sea. These master variables control the oceans’ physical, chemical, and biological processes, such as stratification, circulation, oxygen solubility, ice melt, sea level rise, and redox processes. In the end, our coral reefs, fisheries, and ecosystems are at risk (Figure 10).

Our basic conundrum is that too many humans and polluting technologies affect too many natural resources at larger and larger scales in space and time. Early in the industrial revolution, our problems were local. When soils were depleted, we moved to a different location. When air and water were polluted, we relocated upwind and upstream. As problems grew to the regional scale, they crossed national lines, like acid rain and transboundary air pollutants, so we attempted to pass national laws and international agreements (soft law) to forestall the issues. However, now, problems like climate change have grown to global scale, and it remains to be seen whether international agreements and soft policy incentives can stave off global effects that exceed the limits of our planetary boundaries.

Despite the Paris Agreement of 2015 and subsequent meetings, our emissions of greenhouse gases have failed to decline. Progress on transitioning from fossil fuels to renewable energy like wind and solar power is encouraging, but it is not happening nearly fast enough to stave off the mounting effects of climate change on land and sea. We must decrease emissions to net-zero by 2050 to avoid global warming of 2 °C greater than in preindustrial times. And we must accomplish the task while simultaneously helping the most vulnerable nations and disadvantaged peoples to a path toward better health, education, and welfare. It is truly the most significant challenge of our time.

Investing in renewable energy, green processes and materials, water reuse, biorefineries, a circular economy, regenerative agriculture, reforestation, weatherization of our homes, and electric vehicles and battery storage will provide quality jobs, wealth, and prosperity for future generations while securing a sustainable future. In addition, we must mitigate greenhouse gas emissions while, at the same time, adapting to future changes in climate that are unavoidable. Because of the long time lag in our climate system, it will take decades to stabilize atmospheric chemistry and climate even after we reach net-zero emissions, and we have learned that ocean residence times require decades and longer to cool, degas excess CO_2_, and neutralize acidification.

The good news is that most climatic and oceanic effects discussed in this review are reversible with mitigation measures. However, the loss of species and biodiversity is tragically not reversible–it is permanent. So we applaud the recent agreement by more than 190 countries at the Kunming-Montreal Global Biodiversity Framework to halt and reverse biodiversity loss by the end of this decade by preserving 30% of land and 30% of oceans. Currently, only 17% of land and 10% of oceans are considered protected, so it will be a monumental achievement if humanity can weave together and enlarge the protection of land and marine species. It provides hope for addressing the thorniest environmental problems on earth. We depend on environmental scientists to lead the way in explaining the urgency of action, and it is hoped that this review will provide a small contribution. The future of ecosystems and humans remains in the balance.

## Vocabulary

The ocean’s master variables: Temperature, pH, and pε. Initially emphasized by Lars Gunnar Sillén. These variables control all the state variables of activities and concentrations in ocean chemistry. However, human activities, including the release of greenhouse gases and nitrogen fertilizers, have disrupted these variables, thus affecting critical physical, chemical, and biological reactions in the sea
Greenhouse gases: Those gaseous chemicals which are long-lived in the atmosphere and absorb infrared (longwave) radiation. Such chemicals with two or more atoms per molecule can vibrate and rotate to absorb the earth’s back-radiation and prevent the radiative heat from escaping into deep space. It Is analogous to wrapping a blanket around the atmosphere and keeping the heat energy from escaping
Haber-Bosch process: Developed in the early 1900s for converting nonreactive nitrogen gas into ammonia for fertilizers and other uses. It completely transformed the nitrogen balance on Earth. Not only does the process account for a huge amount of man-made reactive nitrogen in the form of fertilizer ammonia, but it also consumes a significant quantity of natural gas and emits carbon dioxide GHGs
Ocean acidification: Human activity has increased atmospheric carbon dioxide. Increased dissolved carbon dioxide acidifies the ocean, changing in steady state from pH 8.2 to 8.05 today. This drop in pH is causing difficulty for marine organisms to form calcium carbonate shells and skeletons. As a result, oceans will continue to acidify in the future due to CO_2_ emissions, jeopardizing the entire marine food chain, unless fossil fuel burning ceases, and then Earth could begin to return to its original steady state
Oxidation–reduction potential (ORP): ORP is an electrode potential measurement in millivolts, and it is an indication of the tendency of the water to be oxidizing or reducing. A positive ORP indicates oxidizing conditions; a negative ORP implies reducing conditions (the tendency to reduce an oxidized substance). Declining dissolved oxygen concentrations due to human activity cause ORP of the ocean to decrease, indicating more reducing conditions. In this case, reduced compounds like NH_4_^+^, Fe^2+^, CH_4_, HS^–,^ and N_2_O could become more predominant, and ecosystems and fisheries could suffer, much as they do in coastal hypoxia
pε: pε is the negative logarithm of the electron activity in solution. That is, pε = −log{e^–^}. It is a master variable affecting oxidation–reduction reactions in water. Free electrons do not exist in solution, but pε is a useful construct for quantifying the chemical thermodynamic tendency for reduction or oxidation of substances. High electron activity represents reducing conditions, and low electron activity indicates oxidizing conditions
